# The B-Box Family Gene *STO* (*BBX24*) in *Arabidopsis thaliana* Regulates Flowering Time in Different Pathways

**DOI:** 10.1371/journal.pone.0087544

**Published:** 2014-02-03

**Authors:** Feng Li, Jinjing Sun, Donghui Wang, Shunong Bai, Adrian K. Clarke, Magnus Holm

**Affiliations:** 1 Peking University-Yale Joint Research Center of Agricultural and Plant Molecular Biology, National Key Laboratory of Protein Engineering and Plant Gene Engineering, College of Life Sciences, Peking University, Beijing, China; 2 Department of Biological and Environmental Sciences, Gothenburg University, Gothenburg, Sweden; Temasek Life Sciences Laboratory & National University of Singapore, Singapore

## Abstract

Flowering at the appropriate time is crucial for reproductive success and is strongly influenced by various pathways such as photoperiod, circadian clock, *FRIGIDA* and vernalization. Although each separate pathway has been extensively studied, much less is known about the interactions between them. In this study we have investigated the relationship between the photoperiod/circadian clock gene and *FRIGIDA/FLC* by characterizing the function of the B-box *STO* gene family. STO has two B-box Zn-finger domains but lacks the CCT domain. Its expression is controlled by circadian rhythm and is affected by environmental factors and phytohormones. Loss and gain of function mutants show diversiform phenotypes from seed germination to flowering. The *sto-1* mutant flowers later than the wild type (WT) under short day growth conditions, while over-expression of *STO* causes early flowering both in long and short days. *STO* over-expression not only reduces *FLC* expression level but it also activates *FT* and *SOC1* expression. It also does not rely on the other B-box gene *CO* or change the circadian clock system to activate *FT* and *SOC1*. Furthermore, the *STO* activation of *FT* and *SOC1* expression is independent of the repression of *FLC*; rather *STO* and *FLC* compete with each other to regulate downstream genes. Our results indicate that photoperiod and the circadian clock pathway gene *STO* can affect the key flowering time genes *FLC* and *FT/SOC1* separately, and reveals a novel perspective to the mechanism of flowering regulation.

## Introduction

There are several key developmental changes during the plant lifecycle. One of these is flowering, the correct timing of which is critical for reproductive success [1]. Physiological and genetic studies have shown that multiple pathways can promote or repress flowering [1] [2] [3]. The floral pathway integrators *FLOWERING LOCUS C* (*FLC*), *FLOWERING LOCUS T* (*FT*), *SUPPRESSOR OF CONSTANS1* (*SOC1*) and *LEAFY* (*LFY*) are all involved in this transition. The MADS box gene *FLC* is a central repressor of flowering in *Arabidopsis* [4] [5], and its expression is regulated by vernalization and an autonomous pathway involved in chromatin regulation, transcription level and co-transcriptional RNA metabolism [6]. Vernalization pathway genes (*VRN1*, *VRN2*, *VIN3*, *VIL1/VRN5*, *atPRMT5*) repress *FLC* expression by histone modification of *FLC* during and after the cold treatment [7] [8] [9] [10] [11] [12].

*Arabidopsis* has two antagonistic pathways that regulate *FLC* expression. The *FRIGIDA* (*FRI*) pathway is a positive regulator, while a group of genes that belong to the autonomous floral-promotion pathway are negative regulators (i.e., *LD*, *FLD*, *FCA*, *FY*, *FVE*). *FRI* is a unique plant gene that encodes a nuclear-localized protein with a coiled-coil domain [13]. The functional allele of *FRI* is only found in the winter-annual *Arabidopsis*, which requires vernalization to flower rapidly in the spring through repression of *FLC*. In rapid-cycling *Arabidopsis* there is no functional *FRI*; *FLC* expression is kept at low levels and the photoperiod pathway accelerates flowering. *FLC* directly binds and represses the two important flowering genes *FT* and *SOC1* [14]. *FT* is the “florigen” acting as a long distance signal that is transported from leaves to the shoot meristem [15] [16] [17] [18]. The MADS-box gene *SOC1* was initially cloned as a suppressor of the *CONSTANS1* (*CO*) overexpressor [19] [20]. *SOC1* regulation integrates inputs from multiple flowering pathways including photoperiod, vernalization, aging and GA [12] [21]. Besides GA, other phytohormones (BR, ethylene and ABA) also play important roles in flowering, but the underlying mechanisms are less well understood [22] [23] [24]. *SOC1* can also be considered as a meristem-identity gene because it maintains the meristem in a floral state [25]. Both *FT* and *SOC1* are regulated by different flowering pathways [20] [26]. The effect of day length within the photoperiod pathway has been extensively studied [21] [27].

*Arabidopsis* senses changes in day length by the circadian clock, which in turn regulates the transcription factor *CO*. CO belongs to a subfamily of the zinc finger protein family that is now known as the B-Box Zinc Finger Family (BBX) [28]. This family consists of 32 genes divided into five structural groups from I to V. Proteins in group I (including CO) all contain a B-box B1, a B-box B2, and a C-terminal CCT (*CO/COL/TOC1*) domain. Group II members are similar to group I, and contain both B1, B2 and CCT domains, but have minor differences in their B2 domains. Proteins from group III only contain a B1 and CCT domain, while group IV members have B-box B1 and B2 domains but lack the CCT domain (including STO, STH, STH2 and STH3). Proteins in group V have only a single B1 domain [28].The temporal and spatial regulation of *CO* on a transcriptional and protein stability level is the most important element of the photoperiod pathway. Under long day conditions (LD), *CO* transcription is regulated by the circadian clock and accumulates late in the day. *FLAVIN-BINDING*, *KELCH REPEAT*, *F-BOX 1* (*FKF1*) combined with *GIGANTEA (GI)* and *CYCLING DOF FACTOR1* (*CDF1*) not only promote *CO* expression but also stabilize the CO protein in the afternoon in LD [29] [30] [31]. During the night, the CO protein is degraded by COP1 [32]. Based on the complex regulation of *CO*, CO protein accumulates at the end of the day and activates the downstream genes such as *FT* and *SOC1* to promote flowering in LD. In contrast, the CO protein is not stably produced under short day (SD) conditions [21].

The flowering-time integrators *FT* and *SOC1* are common targets of distinct pathways, but the relationship between the different pathways is still unclear. There is little evidence for a direct connection between *FRI*/*FLC* and the photoperiod/circadian clock pathway in flowering time except that they antagonistically regulate common gene targets (*FT* and *SOC1*). In this article, we have studied the function of another B-box family gene *Salt Tolerance* (*STO* or *BBX24*), and show that *STO* (*BBX24*) links the *FRI/FLC* and photoperiod/circadian clock pathways.

## Materials and Methods

### Growth conditions and plant material

Plants were grown in soil under controlled conditions of LD (16 h light/8 h dark) or SD (8 h light/16 h dark) at 22°C. The level of photosynthetic active radiation was 60 µmol photons m^−2^ s^−1^ under both LD and SD conditions. Plants were grown on MS plates, 4 d in dark at 4°C before moved to LD or SD at 22°C. The Columbia (Col-0) ecotype was used. In the *STO* over-expression studies, phenotypic analysis of all transgenic lines and controls were conducted on plates with MS with addition of 50 µg ml^−1^ kanamycin for transgenic plant selection. The mutant seeds are listed in Table S1.

### Measurement of hypocotyl length

Seeds were sterilized in 70% ethanol and 0.01% Triton X-100 for 15 min, followed by 95% ethanol for 10 min. After sterilization, seeds were suspended in 0.1% low-melting-point agarose and spotted on plates containing MS medium (Gibco/BRL) and 0.8% phytagar (Gibco/BRL). Seeds on plates were then stratified in the dark at 4°C for 4 d. Plants were transferred to 22°C. Hypocotyl lengths from 20 seedlings were measured on day 4 with NIH Image 1.62.

### Flowering time determination

Flowering time was determined by counting the number of rosette leaves after bolting. Data were reported as mean leaf number (± S.D.) and were measured from homozygous lines.

### Brassinolide (BR) treatment

Plants were grown on the 1/2 MS plates for 5 d with 0–1000 nM brassinolide (BL), which is the most biologically active BR before measuring hypocotyl length. For the RNA extractions, all seedlings were grown on 1/2 MS plates and treated with 100 nM BL for 3 h or 4 d prior to harvest, with all samples harvested at the same time.

### Ethylene triple response

Dark-grown WT and *sto-1* seedlings were treated with 0–10 µM ACC (the precursor of ethylene) for 3.5 d. SE values were determined from 20 to 30 seedlings.

### Vernalization treatment

Seeds were germinated on agar plates for 4 d at 22°C and vernalized for 4 weeks at 4°C under SD (8 h light/16 h dark). Post vernalization samples continued to grow on agar plates at 22°C under SD.

### RNA extraction and quantitative real-time PCR

Total RNA was isolated from seedlings (4, 8 and 10 d old) or mature rosette leaves using the RNAeasy extraction kit (Qiagen). First-strand cDNA synthesis was performed on 1–2 µg of RNA using the M-MLV System for RT-PCR (Fermentas) followed by PCR amplification with dream Taq DNA Polymerase (Fermentas) or by quantitative PCR (Bio-Rad). Reactions were performed using the primers described in Table S2. For cDNA synthesis, the poly-dT primer was used. The quantitative real-time PCRs were performed with at least three independent RNA samples. For *STO* expression analysis by real-time PCR, all the samples were harvested 6 h into the photoperiod when the peak level of *STO* expression is reached.

### DNA extraction

Plant tissue (200 mg) was ground to a fine paste in 500 µl of CTAB buffer. The CTAB/plant extract mixture was transferred to a microfuge tube and incubated for 15 min at 55°C in a recirculating water bath. After incubation, the CTAB/plant extract mixture was centrifuged at 12000 *g* for 5 min to pellet cell debris. The supernatant was transferred to clean microfuge tubes and 250 µl of chloroform/isoamylalcohol (24∶1) was added to each, and then mixed by inversion. After mixing, tubes were centrifuged at 13000 *g* for 10 min. The upper aqueous phase containing the DNA was transferred to a clean microfuge tube and placed at −20°C for 1 h after the addition of ethanol to precipitate DNA. The precipitated DNA was then pelleted, washed twice in 70% ethanol and then resuspended in sterile DNase-free water.

## Results

### Developmental phenotypes of the *STO* loss- and gain-of-function mutants

**Figure 1:**
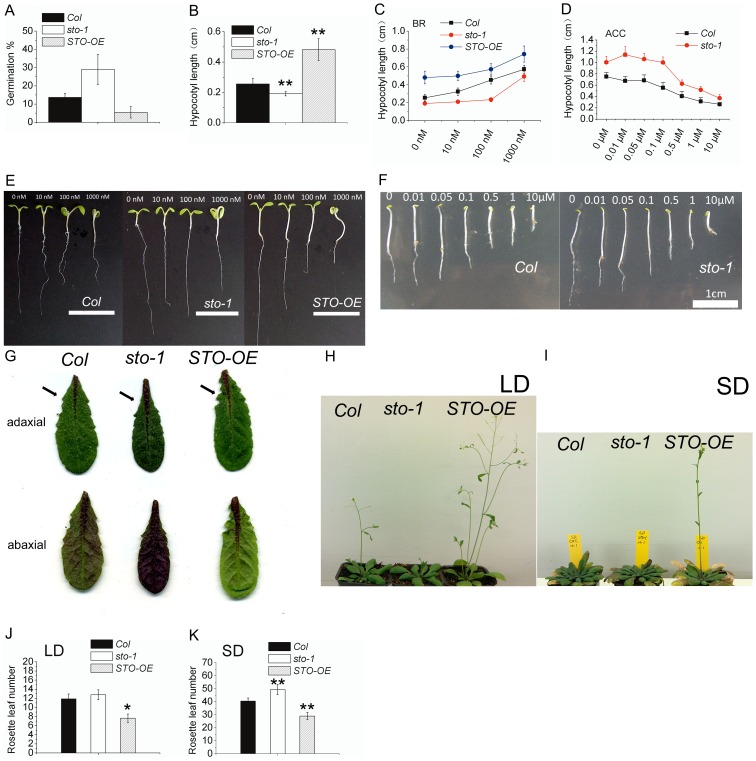
*sto-1* and *STO-OE* show diverse phenotypes. Col, *sto-1* and *STO-OE* were grown under SD conditions and different growth characteristics analyzed. (A) Seed germination as a percentage of total seeds analyzed; (B) hypocotyl length; (C) and (E) Seedling growth as measured by average hypocotyl length (*n* = 20–30) after 5 d on1/2 MS plates treated with BL (0–1000 nM) in SD; (D) and (F) Seedling growth as measured by average hypocotyl length (*n* = 20–30) after treatment with ACC (0–10 µM) for 3.5 days in darkness; (G) Appearance of adult rosette leaves on both adaxial and abaxial surfaces, with arrows indicating the position of leaf serration; (H–K) Flowering time of Col, *sto-1* and *STO-OE* in LD and SD conditions. The average number of rosette leaves in LD (J) and SD (K) was also calculated (*n* = 15–28, * means p<0.05 and ** means p<0.01 in TTEST).

*STO* was originally found to increase the tolerance of yeast to both Li and Na ions [33]. STO, along with STH (*BBX25*) interacts with the WD40 domain of COP1 [34], with *COP1* repressing the transcription of *STO* and contributing to STO protein destabilization in etiolated seedlings [35]. Overall, *STO* acts as a negative regulator in the early photomorphogenesis response to red, far-red, blue and UV-B light signaling [32] [35] [36]. To further uncover the biological function of *STO*, we examined the phenotypes of the *sto-1* T-DNA insertion knockout mutant (SALK_067473) and *STO* over-expression line *STO-OE* (*35s:: STO*) at different developmental stages. Early in development, *STO* appears to repress the rate of seed germination (24 h after transfer to light), with the *sto-1* mutant exhibiting faster and the *STO-OE* line slower germination rates than the WT (Figure 1A). Despite this variation, 90–100% of seeds for all lines studied germinated after 48 hours. At the seedling stage, *sto-1* and *STO-OE* hypocotyls were shorter and longer than WT, respectively (Figure 1B). The difference in hypocotyl length between *sto-1* and *STO-OE* appears due to elongation post germination rather than to the rate of germination itself, and *STO* plays an important role in this process. Moreover, both the *sto-1* and *STO-OE* lines lost sensitivity to low and moderate concentrations of BL (0–100 nM), but all responded to a higher concentration (1000 nM) (Figure 1C and E). Under dark conditions, *sto-1* had slightly longer hypocotyls than the WT and was less sensitive to low concentrations of ACC (0–0.1 µM) (the precursor of ethylene). However, *sto-1* still showed a triple-response phenotype at higher concentrations (Figure 1D and F; it should be noted that the *STO-OE* germination rate was very poor in darkness and ACC treatments). In the adult rosette leaves, *sto-1* accumulated much more anthocyanin on the abaxial surface than WT, while *STO-OE* had much less anthocyanin than WT. In *STO-OE*, the purple color concentrated in the main vascular tissue, while the remaining leaf blade was much greener than WT. *STO* affects not only mature rosette leaf pigmentation but also leaf morphology. In *sto-1*, adult leaves were narrower and more curled than WT, but less serrated, whereas the STO-OE leaves had much deeper serrations than WT (Figure 1G).

In addition to changes in leaf characteristics, flowering time was also affected by *STO* in both LD and SD conditions (Figure 1H–K). Over-expression of *STO* promoted flowering in both LD (Figure 1H and J) and SD (Figure 1I and K) while loss of *STO* delayed flowering relative to the WT but only in SD; both *sto-1* and WT were almost bolting at the same time in LD. These results confirm that *STO* is an important gene affecting different developmental stages throughout the plant lifecycle.

### *STO* expression has diurnal transcript characteristics and is affected by environmental factors and phytohormones

**Figure 2:**
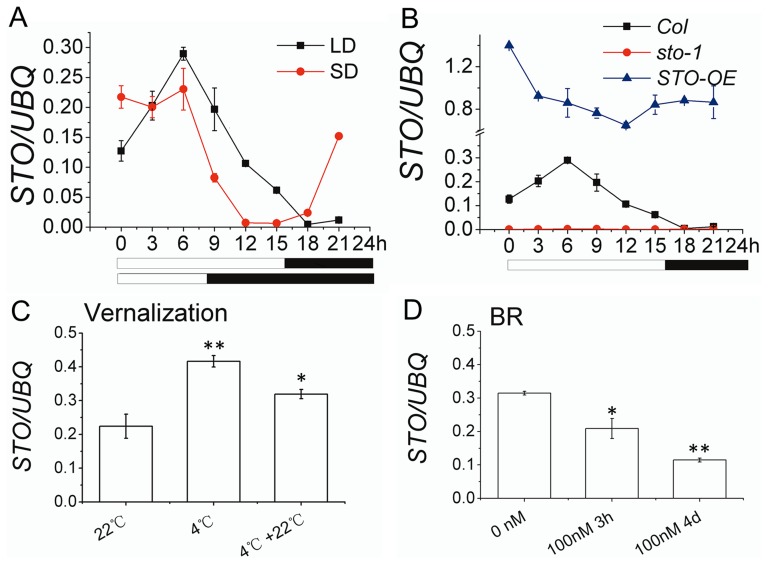
*STO* is regulated by environmental factors and phytohormones. (A) The diurnal expression pattern of *STO* under both LD (16 h light/8 h dark) and SD (8 h light/16 h dark) conditions in eight-day-old seedlings. (B) The diurnal expression pattern of *STO* in eight-day-old seedlings of Col, *sto-1* and *STO-O*E in LD. (C) The effect of vernalization on *STO* expression. Seedlings were either warm-treated (22°C, 2 d, in SD), cold-treated (4°C, 4 weeks, in SD) or cold-treated (4°C, 4 weeks, in SD) and then warm-treated (22°C, 2 d, in SD). (D) The effect of BR on *STO* expression. Seedlings were treated with BL (100 nM) for either 3 h or 4 d. All experiments were performed with two (A–B) or three (C–D) independent replicates, *UBQ10* used as a control. White and black bars represent the light and dark periods, respectively. * means p<0.05 and ** means p<0.01 in TTEST.

To gain insights into the biological function of *STO*, its spatial and temporal expression characteristics were analyzed by qPCR. *STO* is expressed in major tissues of *Arabidopsis* and the highest expression level was found in the shoot apex (Figure S4). Previous studies have shown that expression of *STO* is under circadian clock control [36].The *STO* transcript has diurnal characteristics in both LD and SD conditions (Figure 2A). The highest *STO* expression level was at 6 h into the photoperiod under both LD and SD conditions, after which a rapid decline in LD and SD was observed. During the dark period, however, *STO* expression increased earlier in SD than LD. In the *STO-OE* line, *STO* expression was maintained at high levels in both light and dark conditions (Figure 2B). *STO* expression in WT was also up-regulated by exposure to low temperature (Figure 2C), similar to that previously shown for UV-B treatment [32]. This cold-induced increase in *STO* transcripts, however, was reversed upon returning the plants to the standard growth temperature of 22°C, dropping almost to the levels observed in the control plants (Figure 2C). In contrast, *STO* expression was repressed by treatment with the phytohormone BR (Figure 2D). BR is an essential hormone that regulates a wide range of developmental and physiological processes including cell expansion, vascular differentiation, etiolation, flowering and male fertility [37]. Light and BR antagonistically regulate the developmental process in de-etiolation of plants [38]. When seedlings were grown in 1/2 MS medium and treated with BL (100 nM) for 3 h and 4 d, respectively, *STO* expression was repressed during both exposure times (Figure 2D). Taken together, these phenotypes and expression characteristics indicate that *STO* is an important gene during the development of the plant and its response to certain environmental cues.

### *STO* represses *FLC* expression

Although the role of *STO* in early photomorphogenesis has been well studied [32] [35] [36], little is known about its function during the late developmental stages. Earlier microarray analysis revealed that the level of *FLC* expression increases in *sto-1* etiolated seedlings (unpublished data), results which were confirmed in this study by qPCR at different time points in etiolated seedlings (Figure S1). Both *sto-1* and *STO-OE* have flowering phenotypes; *FLC* was selected for further investigation to examine how this B box family gene affects *Arabidopsis* flowering time.

**Figure 3:**
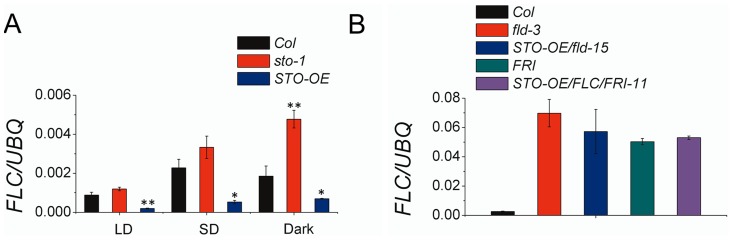
*STO* represses the level of *FLC* expression. (A) Expression of *FLC* in four-day-old seedlings of Col, *sto-1* and *STO-OE* grown in either LD, SD or darkness. (B) Changes in *FLC e*xpression in four-day-old seedlings in SD when *STO* is over-expressed in the *FRI* or *fld-3* background. The level of *FLC* expression in Col and the *FRI* and *fld-3* lines was also included for comparison. All experiments were performed with at least three independent biological replicates, with *UBQ10*used as a control. * means p<0.05 and ** means p<0.01 in TTEST.

We investigated *FLC* expression in different lines and light conditions (i.e., LD, SD, darkness) (Figure 3A). We found that over-expression of *STO* strongly repressed *FLC* expression in all three light conditions, whereas *FLC* expression in *sto-1* seedlings was higher than in WT Col-0 and *STO-OE* in SD and darkness. In the LD condition, however, the level of *FLC* expression in *sto-1* was similar to that in WT seedlings. Moreover, over-expression of *STO* in *FRIGIDA* and *fld-3*, which have high levels of *FLC* transcripts, did not further repress *FLC* expression (Figure 3B). At same time, in *FRI* and *flc-3/FRI* background, *STO* expression level did not show any statistically significant difference compared with WT (Figure S2). These results suggest *STO* is an upstream regulator that represses *FLC* expression.

### *STO* promotes *FT* and *SOC1* expression

In addition to *FRI* and *FLC*, the circadian clock system, photoperiod pathway (*CO*), and flowering integrators (*FT*, *SOC1*) also play important roles in the transition from vegetative to reproductive phases. The circadian clock consists of at least three interlocked transcriptional feedback loops. The LATE ELONGATED HYPOCOTYL (LHY) and CIRCADIAN CLOCK–ASSOCIATED1 (CCA1) proteins are important components of the circadian clock system and have partially redundant functions [39] [40]. *LHY* and *CCA1* not only repress the floral transition under LD and SD conditions, they also accelerate flowering in continuous light by promoting *FT* expression [41]. *FT* and *SOC1* are both *CO* target genes. *CO*, *FT* and *SOC1* are deeply affected by the circadian clock system at different levels [40].

**Figure 4:**
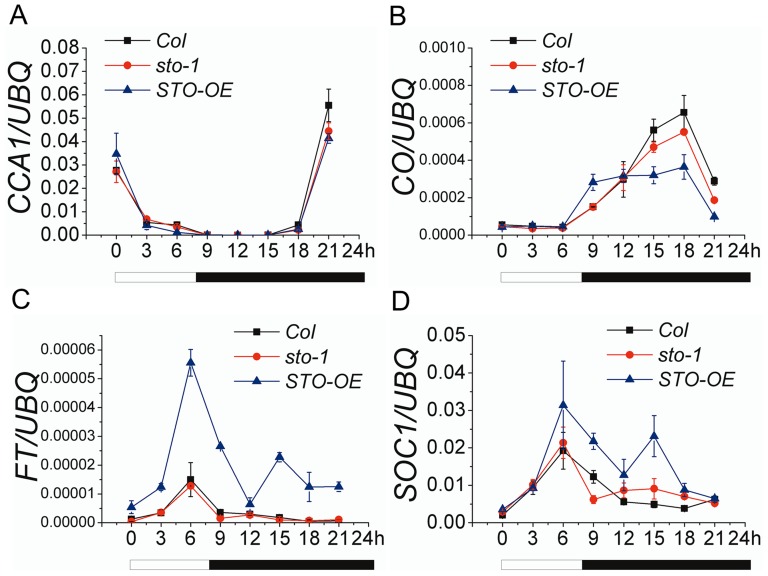
Over-expression of *STO* promotes *FT* and *SOC1* expression independent of *CO* or *CCA1.* The level of *CCA1* (A), *CO* (B), *FT* (C) and *SOC1* (D) expression in eight-day-old seedlings under SD conditions. Data from two independent replicates are shown, with *UBQ10* used as a control. White and black bars represent the light and dark periods, respectively.

To further investigate how *STO* regulates flowering time, we next examined how it affects expression of the aforementioned genes. Several different time points under the SD condition, where the *sto-1* knock-out mutant and *STO* over-expressor showed significant differences in flowering time were investigated (Figures 1 H–K). *CCA1* expression level did not change either in amount or rhythm in Col, *sto-1* and *STO-OE* (Figure 4A). In *sto-1*, *CO* expression level was equal to that in Col during most time points and slightly lower in the dark (15–18 h). In the *STO-OE* line, *CO* expression level was higher compared to that in Col and *sto-1* at the end of the day period (after 6–8 h) but lower during the dark (Figure 4B). The expression level of *FT* and *SOC1* first peaked in the middle of the photoperiod (6 h) and then again in the dark (15 h) (Figures 4C and D). Since both CO and STO proteins are degraded by COP1 in the dark [32] [34] [42], the second peak of *FT* and *SOC1* expression in *STO-OE* might be a result of the extra STO protein overwhelming the ability of COP1 to degrade it, thereby activating *FT* and *SOC1* expression. Also in the *STO-OE* line, *FT* and *SOC1* expression increased earlier than *CO* and started to decrease at the time when *CO* had only just begun to increase (i.e., after 6 h; Figures 4C and D). These results suggest that *STO* activates *FT* and *SOC1* expression in a *CO* and circadian rhythm-independent manner, although this does not exclude the possibility that *CO* affects *STO* at the protein level. In the *STO-OE* line, moreover, the promotion of *FT* expression mainly occurred in cotyledons, whereas the expression of *FT* in *sto-1* cotyledons was lower than in Col. Furthermore, the level of *FT* expression did not differ in the shoot apex, hypocotyl and root (Figure S5).

### *STO* competes with *FLC* to promote *FT* and *SOC1* expression

**Figure 5:**
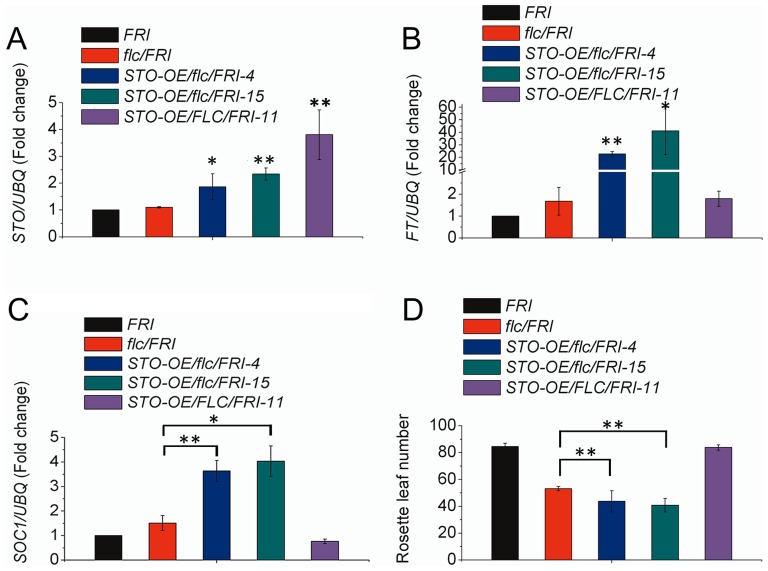
Over-expression of *STO* promotes *FT* and *SOC1* expression independent of *FLC* repression. The level of *STO* (A), *FT* (B) and *SOC1* (C) expression in eight-day-old seedlings of the indicated genotypes. In the analysis, the data from STO-OE/flc/FRI-4 and -15 were compared to that of flc/FRI, while the data for STO-OE/FLC/FRI-11 was compared to that of FRI. Data from three independent replicates are shown, with *UBQ10* used as a control. (D) The number of rosette leaves in each indicated genotype at the time of flowering. All seedlings and mature plants were grown in SD. ** means p<0.01 in TTEST.

Since *FLC* is known to block *FT* by directly binding to its chromatin [14], there are two possible explanations why *STO* can promote flowering: that either *STO* directly represses *FLC* and activates *FT* and *SOC1* expression simultaneously, or that *STO* only represses *FLC* that then leads to the activation of both *FT* and *SOC1*. To investigate these two scenarios, we overexpressed *STO* in different genetic backgrounds (Figure S3) to hopefully reveal the regulatory relationship between *STO* and *FLC* (Figure 5A). In the *flc-3/FRI* lines, which lack full length *FLC* but have functional *FRI*, *FT* and *SOC1* expression was slightly higher than in *FLC*/*FRI* under SD conditions. In the *STO-OE*/*flc-3/FRI* lines (individual lines 4 and 15), *FT* and *SOC1* expression significantly increased (Figures 5B and C), showing that *STO* activates *FT* and *SOC1* expression independently of the repression of *FLC*. If *STO* activates *FT* and *SOC1* expression by only reducing *FLC* expression, there should be similar amounts of *FT* and *SOC1* transcripts in both the *STO-OE*/*flc-3/FRI* and *flc-3/FRI* lines. Moreover, these two lines (*STO*/*flc-3/FRI* -4 and 15) also flowered earlier under SD conditions (Figure 5D and Table S 3). In contrast, in the *STO-OE/FLC/FRI* line (line11) that has high levels of *FLC* and over-expresses *STO*, the level of *FT* expression was only slightly higher than in the *FRI* line, and *SOC1* transcripts were no longer up-regulated (Figures 5B and C).

## Discussion

In this work, we have examined in detail the biological function of *STO* from seed germination to flowering by the use of loss- and gain-of-function mutants. *STO* loss- and gain-of-function mutants both lost sensitivity to low concentrations of phytohormones such as BR and ethylene. Both BR and ethylene affect flowering time. We showed that *STO* was involved in those signaling pathways at least in the seedling stage (Figure 1C–F). In *Arabidopsis*, BR biosynthetic and signaling pathway mutants exhibit delayed flowering phenotypes [43] [44]. BR can promote flowering time by affecting circadian clock and *FLC* at both the transcription and chromatin modification levels [44] [45]. BR and *STO* have similar functions in the repression of photomorphogenesis, increased hypocotyl length and promotion of flowering (Figure 1 and [24]). However, the relationship between *STO* and BR signaling is rather complicated, as *STO* expression was repressed by BL treatment (Figure 2D). The reason for this conflicting observation could be that BL treatment results in accumulation of STO protein and feedback inhibition of *STO* transcription. Another explanation is that they may have common downstream genes, such as *GATA2* and *GATA4*, which are positive regulators of photomorphogenesis and are repressed by BR signaling [38]. *GATA2 and GATA4* expression levels in *sto-1* and *STO-OE* were higher and lower than WT, respectively (unpublished data). BR signaling could through repressing *STO* achieve the appropriate expression level of downstream genes. In addition, *STO* expression was also regulated by environmental factors, such as the photoperiod and cold temperature (Figure 2B and C). All of those environmental and endogenous factors substantially affect plant flowering time.

Important was the observation that over-expression of *STO* produced an early flowering phenotype under both LD and SD conditions, whereas loss of *STO* caused late flowering under SD. Altogether, *STO* appears to function more than just a negative regulator during early photomorphogenesis, and instead to play a crucial role connecting different signaling pathways throughout the plant lifecycle.

The observations from this study support the following conclusions: (i) *STO* represses the flowering repressor *FLC*; (ii) *STO* can up-regulate *FT* and *SOC1* expression, and; (iii) *STO* competes with *FLC* in their regulation of *FT* and *SOC1. STO* can repress *FLC* in LD, SD and darkness. However, in *the FRI* and *fld-3* genetic background, over-expressed *STO* can no longer repress *FLC*. Since *STO* lacks the transcriptional repression/activation domain, it could be that it interacts with another complex to regulate *FLC* expression. *FLC* is not only a repressor of flowering time, but it is also functional throughout the lifecycle of the plant [14], such as promoting seed germination [46], lengthening the circadian period, and vegetative development [47]. Interestingly, the *Ler* background line that has a low-expressing *FLC* allele exhibited high dormancy. A high-expressing *FLC* allele produced a significantly higher germination rate at cool temperatures (10°C), but only slightly higher at warmer temperature (22°C) [46]. We found that the *STO-OE* line which has low *FLC* expression level also displayed slower germination rates, whereas the *sto-1* germination rate was slightly higher than the WT (Figure 1 A, B). As a consequence, we propose that *STO* as a photoperiod/circadian clock controlled gene is involved in the regulation of *FLC*, although the molecular mechanism by which this occurs remains unclear.

We have demonstrated that *STO* activates *FT* and *SOC1* expression, thereby promoting flowering. Given that *STO* expression is controlled by photoperiod/circadian rhythm, it would appear that it is a new component within this regulatory pathway. Moreover, *STO* stimulation of *FT* and *SOC1* expression does not rely on repression of *FLC* but rather *STO* competes with *FLC* to regulate *FT* and *SOC1* expression and thereby promotes flowering. Overall, these characteristics of *STO* reveal new relationships between *FRI/FLC* and the photoperiod/circadian clock pathway.

The combination of phenotype, expression characteristics and genetic results extends our knowledge on the biological function of the B-box zinc finger family in plant development, especially in the transition from vegetative to reproductive phase. Given the central roles of *FLC*, *FT* and *SOC1* in flowering-time regulation in *Arabidopsis*, these findings suggest that the B-box family gene *STO*, which is without the CCT domain, plays an important role in the control of flowering in *Arabidopsis*. The behavior of *STO* in promoting flowering gives us new clues in understanding of how plants integrate environmental and developmental signals. We can therefore expect that the relationship between the different pathways to be more complex than first suspected.

The homologous B-box zinc-finger family genes are widely conserved in higher plants [36] [48] [49]. Despite this, the study of flowering time only focuses on the first group (*CO* and *CO*-like *COL*'S [48]) because previous research has indicated that both B-box motifs and CCT domains are important for promoting flowering under LD conditions [50] [51]. *CO* and *COL* genes have been well studied. While resembling *STO* and *STH1-3*, they also show the opposite function. Flowering in the *CO* mutant is delayed in LD while overexpression of *CO* results in the acceleration of flowering in both LD and SD. Over-expression of *COL5* can induce flowering in SD but *col5* mutants do not show altered flowering [52]. Over-expression of *COL9* resulted in delayed flowering, whereas the *col9* mutant flowered earlier under LD conditions. *COL9* negatively regulates *CO* expression, but it does not appear to directly affect flowering time [53]. Moreover, *CO*, *COL5* and *COL9* influence flowering time mainly through *FT* and *SOC1* and no reports to date have shown that they can affect *FLC* expression. Interestingly, a recent study has shown that *COL1* and -*2*, which are the most closely-related genes to *CO*, do not affect flowering time due to the amino acid differences coded for within the first exon [54]. The first B-box domain may have a more important role than others in affecting flowering time. *COL* genes (including *STO*) have evolved rapidly in the *Brassicaceae* family [48] [55]. It is possible that the effect of *STO* on flowering time independent of the CCT domain is not a unique case. It will be interesting to investigate if other members that lack the CCT domain within the B-box family are also involved in regulating flowering.

## Supporting Information

Figure S1 Confirmation of microarray data. Col, *sto-1* and *STO-OE* were grown in darkness, with *FLC* expression level checked at 2, 4 and 6 d. *FLC* expression levels of the indicated genotypes were checked, and all lines were grown under SD.
(TIF)

Figure S2 *STO* expression levels in different lines. The level of *STO* expression in the indicated genotypes was checked in four-day-old seedlings grown under SD. Data from three independent replicates are shown, with *UBQ10* used as a control.
(TIF)

Figure S3 Double mutant genomic PCR and RT-PCR confirmation. (A) Genomic PCR test was performed in F2 *FRI/flc-3 x STO-OE*, with the lines 4, 7, 10 and 15 being *flc* homozygous. (B) Functional *FRI* allele's have a BsmFI restriction site. Restriction endonuclease BsmFI was used to test *FRI* homozygous in F2 *FRI/flc-3 x STO-OE*, with the lines 4,7,10 and 15 having a functional *FRI*. (C) RT-PCR test of full-length *FLC* expression, with the lines 4, 10 and 15 having no *FLC* mRNA. (D) RT-PCR test of *FLD* expression level in F2 *fld-3 x STO-OE*, with *UBQ10* as the control. The lines 11, 13, 15, 19 and 21 were *fld* homozygous. (E) Restriction endonuclease BsmFI was used to test *FRI* homozygous in F2 *FRI x STO-OE*. The lines 9, 11, 12, 15 and 17 were *FRI* homozygous. All lines were grown for two weeks under SD prior to genomic PCR or RT-PCR.
(TIF)

Figure S4 Determination of *STO* expression levels in different plant tissues. The level of *STO* expression in different tissues of *Arabidopsis* was analyzed in ten-day-old seedlings (cotyledon, shoot apex, hypocotyl and root) and adult plants (rosetta leaf and flower meristem). Data from three or four independent replicates are shown, with *UBQ10* used as a control. * means p<0.05 and ** means p<0.01 in TTEST. Plants were grown under LD.
(TIF)

Figure S5 Increased expression of *FT* in cotyledons and hypocotyls. The level of *FT* expression in different tissues (cotyledon, shoot apex [including young leaf primordial], hypocotyl and root) of ten-day-old seedlings of Col, *sto-1* and *STO-OE*. . Data from three independent replicates are shown, with *UBQ10* used as a control. * means p<0.05 and ** means p<0.01 in TTEST. Plants were grown under LD.
(TIF)

Table S1 Seed list.
(PDF)

Table S2 Primer list.
(PDF)

Table S3 Rosette leaf number at flowering time for the indicated genotypes.
(PDF)

## References

[1] Baurle I, Dean C (2006) The timing of developmental transitions in plants. Cell 125: 655–664. 16713560 10.1016/j.cell.2006.05.005

[2] Bernier G, Havelange A, Houssa C, Petitjean A, Lejeune P (1993) Physiological Signals That Induce Flowering. Plant Cell 5: 1147–1155. 12271018 10.1105/tpc.5.10.1147 PMC160348

[3] Simpson GG, Dean C (2002) Arabidopsis, the Rosetta stone of flowering time? Science 296: 285–289. 11951029 10.1126/science.296.5566.285

[4] Michaels SD, Amasino RM (1999) FLOWERING LOCUS C encodes a novel MADS domain protein that acts as a repressor of flowering. Plant Cell 11: 949–956. 10330478 10.1105/tpc.11.5.949 PMC144226

[5] Sheldon CC, Burn JE, Perez PP, Metzger J, Edwards JA, et al (1999) The FLF MADS box gene: a repressor of flowering in Arabidopsis regulated by vernalization and methylation. Plant Cell 11: 445–458. 10072403 10.1105/tpc.11.3.445 PMC144185

[6] Crevillen P, Dean C (2011) Regulation of the floral repressor gene FLC: the complexity of transcription in a chromatin context. Curr Opin Plant Biol 14: 38–44. 20884277 10.1016/j.pbi.2010.08.015

[7] Henderson IR, Dean C (2004) Control of Arabidopsis flowering: the chill before the bloom. Development 131: 3829–3838. 15289433 10.1242/dev.01294

[8] Bastow R, Mylne JS, Lister C, Lippman Z, Martienssen RA, et al (2004) Vernalization requires epigenetic silencing of FLC by histone methylation. Nature 427: 164–167. 14712277 10.1038/nature02269

[9] Sung S, Schmitz RJ, Amasino RM (2006) A PHD finger protein involved in both the vernalization and photoperiod pathways in Arabidopsis. Genes Dev 20: 3244–3248. 17114575 10.1101/gad.1493306 PMC1686601

[10] Schmitz RJ, Sung S, Amasino RM (2008) Histone arginine methylation is required for vernalization-induced epigenetic silencing of FLC in winter-annual Arabidopsis thaliana. Proc Natl Acad Sci U S A 105: 411–416. 18178621 10.1073/pnas.0710423104 PMC2206549

[11] Sung S, Amasino RM (2004) Vernalization in Arabidopsis thaliana is mediated by the PHD finger protein VIN3. Nature 427: 159–164. 14712276 10.1038/nature02195

[12] Kim DH, Doyle MR, Sung S, Amasino RM (2009) Vernalization: winter and the timing of flowering in plants. Annu Rev Cell Dev Biol 25: 277–299. 19575660 10.1146/annurev.cellbio.042308.113411

[13] Johanson U, West J, Lister C, Michaels S, Amasino R, et al (2000) Molecular analysis of FRIGIDA, a major determinant of natural variation in Arabidopsis flowering time. Science 290: 344–347. 11030654 10.1126/science.290.5490.344

[14] Deng WW, Ying H, Helliwell CA, Taylor JM, Peacock WJ, et al (2011) FLOWERING LOCUS C (FLC) regulates development pathways throughout the life cycle of Arabidopsis. Proceedings of the National Academy of Sciences of the United States of America 108: 6680–6685. 21464308 10.1073/pnas.1103175108 PMC3081018

[15] Kardailsky I, Shukla VK, Ahn JH, Dagenais N, Christensen SK, et al (1999) Activation tagging of the floral inducer FT. Science 286: 1962–1965. 10583961 10.1126/science.286.5446.1962

[16] Kobayashi Y, Kaya H, Goto K, Iwabuchi M, Araki T (1999) A pair of related genes with antagonistic roles in mediating flowering signals. Science 286: 1960–1962. 10583960 10.1126/science.286.5446.1960

[17] Wigge PA, Kim MC, Jaeger KE, Busch W, Schmid M, et al (2005) Integration of spatial and temporal information during floral induction in Arabidopsis. Science 309: 1056–1059. 16099980 10.1126/science.1114358

[18] Mathieu J, Warthmann N, Kuttner F, Schmid M (2007) Export of FT protein from phloem companion cells is sufficient for floral induction in Arabidopsis. Curr Biol 17: 1055–1060. 17540570 10.1016/j.cub.2007.05.009

[19] Lee H, Suh SS, Park E, Cho E, Ahn JH, et al (2000) The AGAMOUS-LIKE 20 MADS domain protein integrates floral inductive pathways in Arabidopsis. Genes Dev 14: 2366–2376. 10995392 10.1101/gad.813600 PMC316936

[20] Samach A, Onouchi H, Gold SE, Ditta GS, Schwarz-Sommer Z, et al (2000) Distinct roles of CONSTANS target genes in reproductive development of Arabidopsis. Science 288: 1613–1616. 10834834 10.1126/science.288.5471.1613

[21] Srikanth A, Schmid M (2011) Regulation of flowering time: all roads lead to Rome. Cellular and Molecular Life Sciences 68: 2013–2037. 21611891 10.1007/s00018-011-0673-y PMC11115107

[22] Achard P, Baghour M, Chapple A, Hedden P, Van Der Straeten D, et al (2007) The plant stress hormone ethylene controls floral transition via DELLA-dependent regulation of floral meristem-identity genes. Proc Natl Acad Sci U S A 104: 6484–6489. 17389366 10.1073/pnas.0610717104 PMC1851083

[23] Kotchoni SO, Larrimore KE, Mukherjee M, Kempinski CF, Barth C (2009) Alterations in the endogenous ascorbic acid content affect flowering time in Arabidopsis. Plant Physiol 149: 803–815. 19028878 10.1104/pp.108.132324 PMC2633856

[24] Li J, Nagpal P, Vitart V, McMorris TC, Chory J (1996) A role for brassinosteroids in light-dependent development of Arabidopsis. Science 272: 398–401. 8602526 10.1126/science.272.5260.398

[25] Melzer S, Lens F, Gennen J, Vanneste S, Rohde A, et al (2008) Flowering-time genes modulate meristem determinacy and growth form in Arabidopsis thaliana. Nat Genet 40: 1489–1492. 18997783 10.1038/ng.253

[26] Amasino RM, Michaels SD (2010) The timing of flowering. Plant Physiol 154: 516–520. 20921176 10.1104/pp.110.161653 PMC2948982

[27] Kobayashi Y, Weigel D (2007) Move on up, it's time for change–mobile signals controlling photoperiod-dependent flowering. Genes Dev 21: 2371–2384. 17908925 10.1101/gad.1589007

[28] Khanna R, Kronmiller B, Maszle DR, Coupland G, Holm M, et al (2009) The Arabidopsis B-box zinc finger family. Plant Cell 21: 3416–3420. 19920209 10.1105/tpc.109.069088 PMC2798317

[29] Imaizumi T, Schultz TF, Harmon FG, Ho LA, Kay SA (2005) FKF1 F-box protein mediates cyclic degradation of a repressor of CONSTANS in Arabidopsis. Science 309: 293–297. 16002617 10.1126/science.1110586

[30] Sawa M, Nusinow DA, Kay SA, Imaizumi T (2007) FKF1 and GIGANTEA complex formation is required for day-length measurement in Arabidopsis. Science 318: 261–265. 17872410 10.1126/science.1146994 PMC3709017

[31] Song YH, Smith RW, To BJ, Millar AJ, Imaizumi T (2012) FKF1 conveys timing information for CONSTANS stabilization in photoperiodic flowering. Science 336: 1045–1049. 22628657 10.1126/science.1219644 PMC3737243

[32] Jiang L, Wang Y, Li QF, Bjorn LO, He JX, et al (2012) Arabidopsis STO/BBX24 negatively regulates UV-B signaling by interacting with COP1 and repressing HY5 transcriptional activity. Cell Res 22: 1046–1057. 22410790 10.1038/cr.2012.34 PMC3367526

[33] Lippuner V, Cyert MS, Gasser CS (1996) Two classes of plant cDNA clones differentially complement yeast calcineurin mutants and increase salt tolerance of wild-type yeast. J Biol Chem 271: 12859–12866. 8662738 10.1074/jbc.271.22.12859

[34] Holm M, Hardtke CS, Gaudet R, Deng XW (2001) Identification of a structural motif that confers specific interaction with the WD40 repeat domain of Arabidopsis COP1. EMBO J 20: 118–127. 11226162 10.1093/emboj/20.1.118 PMC140188

[35] Indorf M, Cordero J, Neuhaus G, Rodriguez-Franco M (2007) Salt tolerance (STO), a stress-related protein, has a major role in light signalling. Plant J 51: 563–574. 17605755 10.1111/j.1365-313X.2007.03162.x

[36] Kumagai T, Ito S, Nakamichi N, Niwa Y, Murakami M, et al (2008) The common function of a novel subfamily of B-Box zinc finger proteins with reference to circadian-associated events in Arabidopsis thaliana. Biosci Biotechnol Biochem 72: 1539–1549. 18540109 10.1271/bbb.80041

[37] Kim TW, Wang ZY (2010) Brassinosteroid signal transduction from receptor kinases to transcription factors. Annu Rev Plant Biol 61: 681–704. 20192752 10.1146/annurev.arplant.043008.092057

[38] Luo XM, Lin WH, Zhu S, Zhu JY, Sun Y, et al (2010) Integration of light- and brassinosteroid-signaling pathways by a GATA transcription factor in Arabidopsis. Dev Cell 19: 872–883. 21145502 10.1016/j.devcel.2010.10.023 PMC3022420

[39] Harmer SL (2009) The circadian system in higher plants. Annu Rev Plant Biol 60: 357–377. 19575587 10.1146/annurev.arplant.043008.092054

[40] Imaizumi T (2010) Arabidopsis circadian clock and photoperiodism: time to think about location. Current Opinion in Plant Biology 13: 83–89. 19836294 10.1016/j.pbi.2009.09.007 PMC2818179

[41] Fujiwara S, Oda A, Yoshida R, Niinuma K, Miyata K, et al (2008) Circadian clock proteins LHY and CCA1 regulate SVP protein accumulation to control flowering in Arabidopsis. Plant Cell 20: 2960–2971. 19011118 10.1105/tpc.108.061531 PMC2613671

[42] Jang S, Marchal V, Panigrahi KC, Wenkel S, Soppe W, et al (2008) Arabidopsis COP1 shapes the temporal pattern of CO accumulation conferring a photoperiodic flowering response. EMBO J 27: 1277–1288. 18388858 10.1038/emboj.2008.68 PMC2291449

[43] Clouse S (1997) Molecular genetic analysis of brassinosteroid action. Plant Physiology 702–709.

[44] Li J, Li Y, Chen S, An L (2010) Involvement of brassinosteroid signals in the floral-induction network of Arabidopsis. J Exp Bot 61: 4221–4230. 20685730 10.1093/jxb/erq241

[45] Domagalska MA, Schomburg FM, Amasino RM, Vierstra RD, Nagy F, et al (2007) Attenuation of brassinosteroid signaling enhances FLC expression and delays flowering. Development 134: 2841–2850. 17611230 10.1242/dev.02866

[46] Chiang GC, Barua D, Kramer EM, Amasino RM, Donohue K (2009) Major flowering time gene, flowering locus C, regulates seed germination in Arabidopsis thaliana. Proc Natl Acad Sci U S A 106: 11661–11666. 19564609 10.1073/pnas.0901367106 PMC2710639

[47] Willmann MR, Poethig RS (2011) The effect of the floral repressor FLC on the timing and progression of vegetative phase change in Arabidopsis. Development 138: 677–685. 21228003 10.1242/dev.057448 PMC3026413

[48] Griffiths S, Dunford RP, Coupland G, Laurie DA (2003) The evolution of CONSTANS-like gene families in barley, rice, and Arabidopsis. Plant Physiol 131: 1855–1867. 12692345 10.1104/pp.102.016188 PMC166942

[49] Almada R, Cabrera N, Casaretto JA, Ruiz-Lara S, Gonzalez Villanueva E (2009) VvCO and VvCOL1, two CONSTANS homologous genes, are regulated during flower induction and dormancy in grapevine buds. Plant Cell Rep 28: 1193–1203. 19495771 10.1007/s00299-009-0720-4

[50] Putterill J, Robson F, Lee K, Simon R, Coupland G (1995) The CONSTANS gene of Arabidopsis promotes flowering and encodes a protein showing similarities to zinc finger transcription factors. Cell 80: 847–857. 7697715 10.1016/0092-8674(95)90288-0

[51] Robson F, Costa MM, Hepworth SR, Vizir I, Pineiro M, et al (2001) Functional importance of conserved domains in the flowering-time gene CONSTANS demonstrated by analysis of mutant alleles and transgenic plants. Plant J 28: 619–631. 11851908 10.1046/j.1365-313x.2001.01163.x

[52] Hassidim M, Harir Y, Yakir E, Kron I, Green RM (2009) Over-expression of CONSTANS-LIKE 5 can induce flowering in short-day grown Arabidopsis. Planta 230: 481–491. 19504268 10.1007/s00425-009-0958-7

[53] Cheng XF, Wang ZY (2005) Overexpression of COL9, a CONSTANS-LIKE gene, delays flowering by reducing expression of CO and FT in Arabidopsis thaliana. Plant J 43: 758–768. 16115071 10.1111/j.1365-313X.2005.02491.x

[54] Kim SK, Park HY, Jang YH, Lee JH, Kim JK (2013) The sequence variation responsible for the functional difference between the CONSTANS protein, and the CONSTANS-like (COL) 1 and COL2 proteins, resides mostly in the region encoded by their first exons. Plant Sci 199–200: 71–78. 10.1016/j.plantsci.2012.09.019 23265320

[55] Lagercrantz U, Axelsson T (2000) Rapid evolution of the family of CONSTANS LIKE genes in plants. Mol Biol Evol 17: 1499–1507. 11018156 10.1093/oxfordjournals.molbev.a026249

