# Supplementary figures and images for: The B-Box Family Gene STO (BBX24) in Arabidopsis thaliana Regulates Flowering Time in Different Pathways
Source: PLoS One. 2014 Feb 3;9(2):e87544. doi: 10.1371/journal.pone.0087544 (PMC3911981; doi:10.1371/journal.pone.0087544)

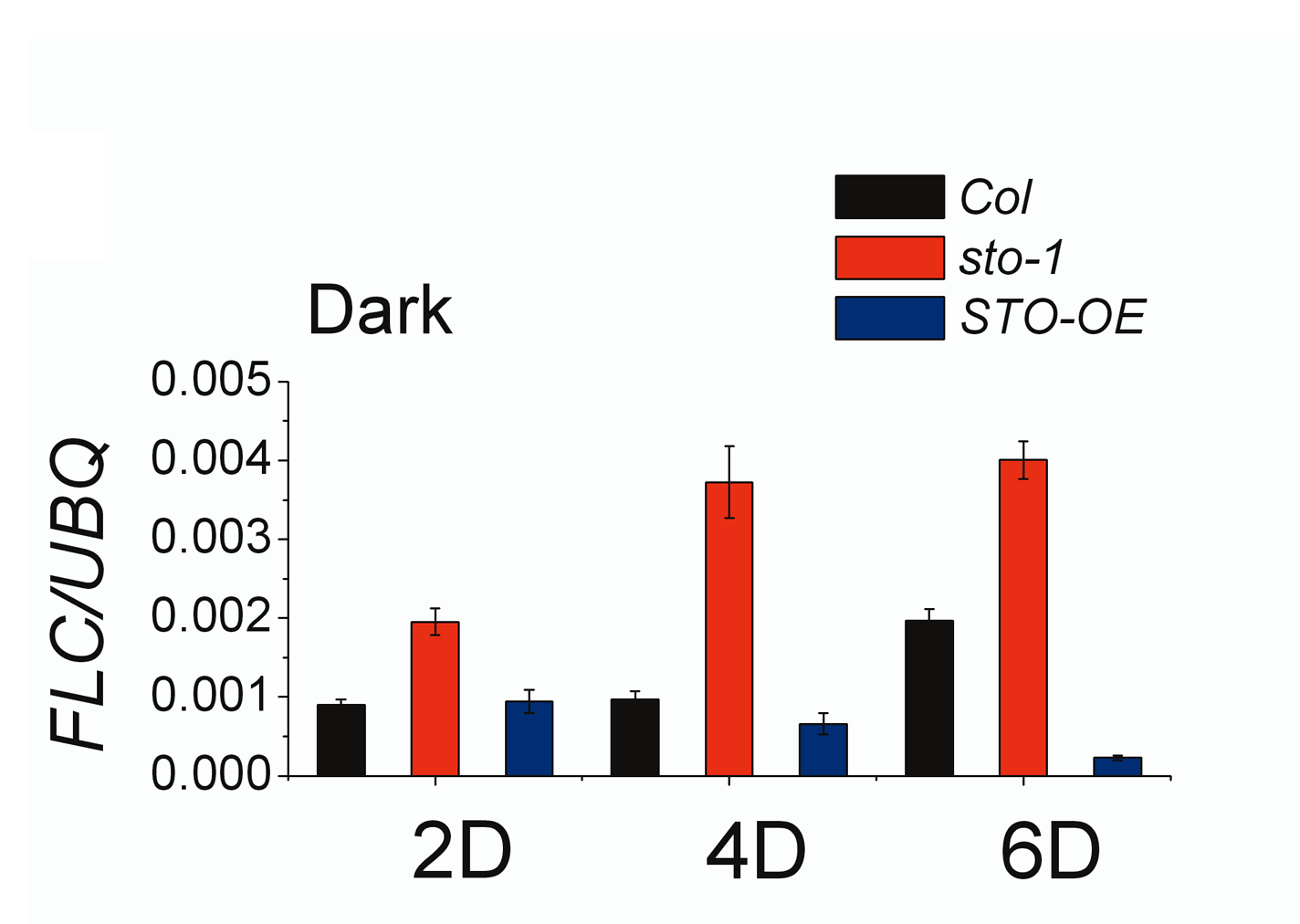

Supplement: Figure S1 — Confirmation of microarray data. Col, sto-1 and STO-OE were grown in darkness, with FLC expression level checked at 2, 4 and 6 d. FLC expression levels of the indicated genotypes were checked, and all lines were grown under SD. (TIF) [file pone.0087544.s001.tif]

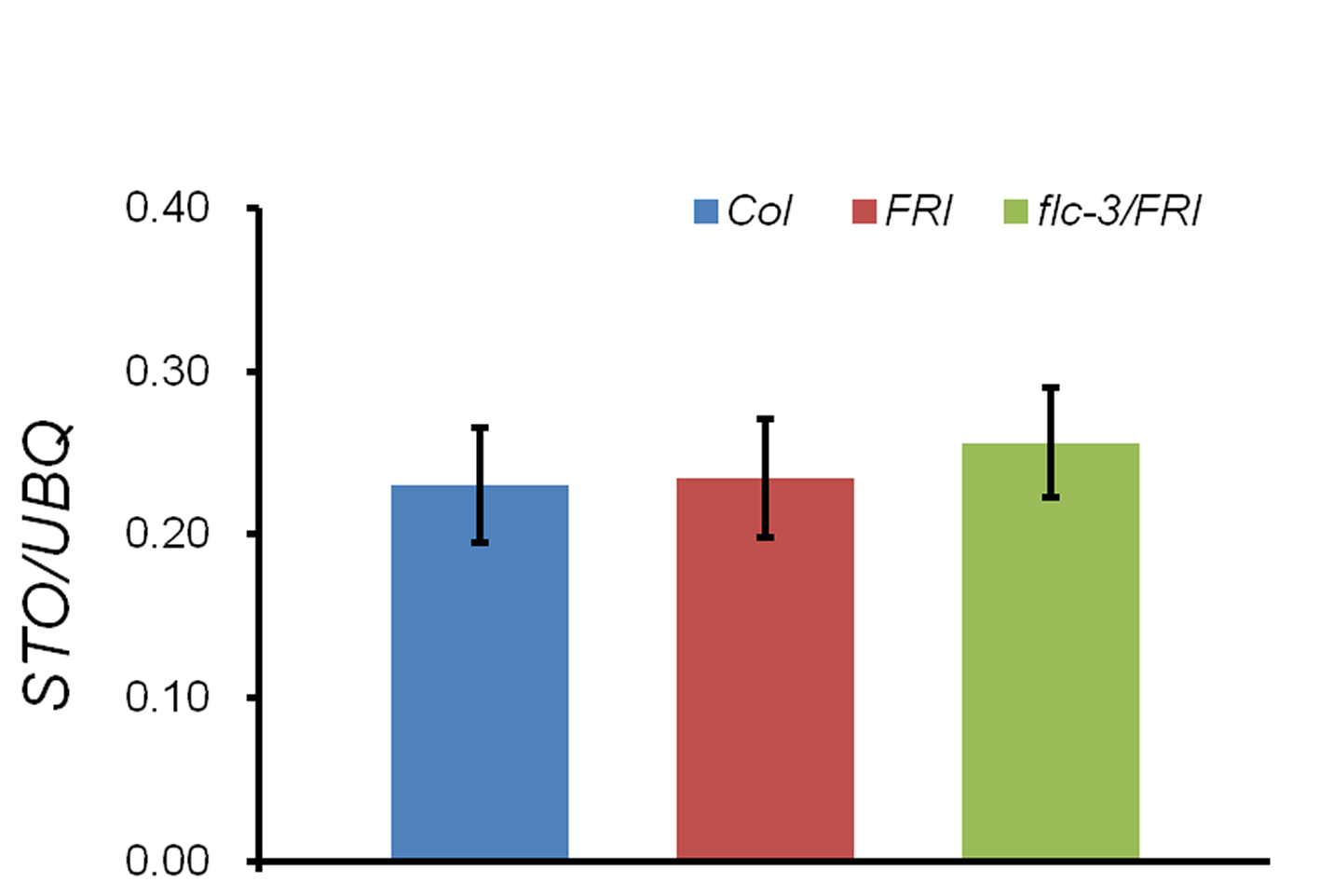

Supplement: Figure S2 — STO expression levels in different lines. The level of STO expression in the indicated genotypes was checked in four-day-old seedlings grown under SD. Data from three independent replicates are shown, with UBQ10 used as a control. (TIF) [file pone.0087544.s002.tif]

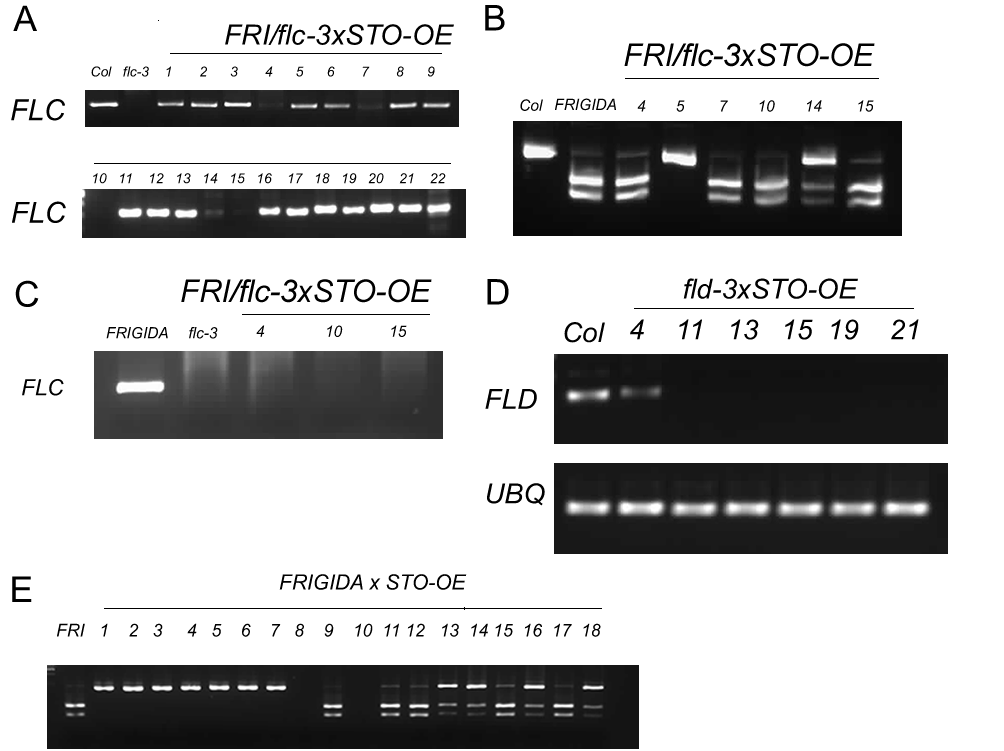

Supplement: Figure S3 — Double mutant genomic PCR and RT-PCR confirmation. (A) Genomic PCR test was performed in F2 FRI/flc-3 x STO-OE, with the lines 4, 7, 10 and 15 being flc homozygous. (B) Functional FRI allele's have a BsmFI restriction site. Restriction endonuclease BsmFI was used to test FRI homozygous in F2 FRI/flc-3 x STO-OE, with the lines 4,7,10 and 15 having a functional FRI. (C) RT-PCR test of full-length FLC expression, with the lines 4, 10 and 15 having no FLC mRNA. (D) RT-PCR test of FLD expression level in F2 fld-3 x STO-OE, with UBQ10 as the control. The lines 11, 13, 15, 19 and 21 were fld homozygous. (E) Restriction endonuclease BsmFI was used to test FRI homozygous in F2 FRI x STO-OE. The lines 9, 11, 12, 15 and 17 were FRI homozygous. All lines were grown for two weeks under SD prior to genomic PCR or RT-PCR. (TIF) [file pone.0087544.s003.tif]

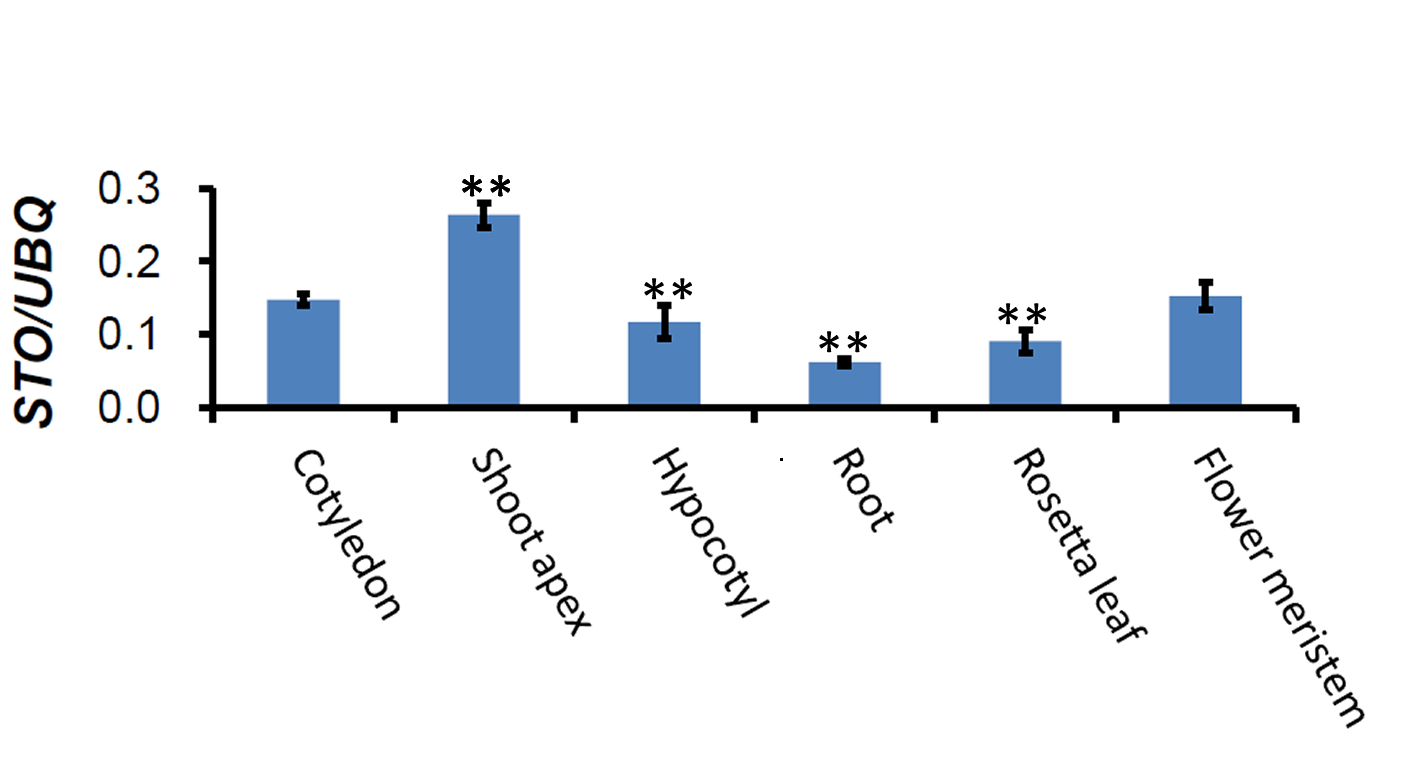

Supplement: Figure S4 — Determination of STO expression levels in different plant tissues. The level of STO expression in different tissues of Arabidopsis was analyzed in ten-day-old seedlings (cotyledon, shoot apex, hypocotyl and root) and adult plants (rosetta leaf and flower meristem). Data from three or four independent replicates are shown, with UBQ10 used as a control. * means p<0.05 and ** means p<0.01 in TTEST. Plants were grown under LD. (TIF) [file pone.0087544.s004.tif]

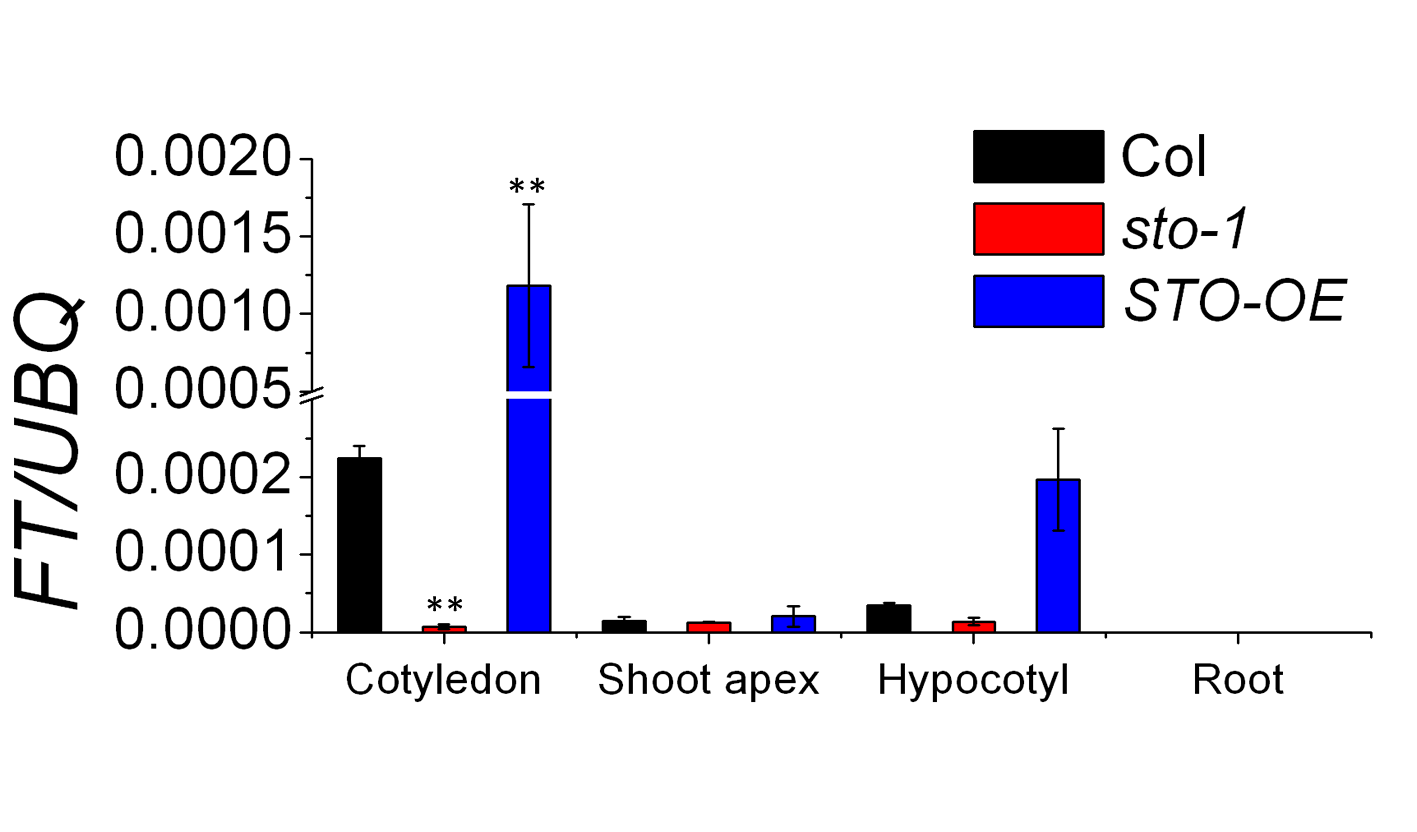

Supplement: Figure S5 — Increased expression of FT in cotyledons and hypocotyls. The level of FT expression in different tissues (cotyledon, shoot apex [including young leaf primordial], hypocotyl and root) of ten-day-old seedlings of Col, sto-1 and STO-OE. . Data from three independent replicates are shown, with UBQ10 used as a control. * means p<0.05 and ** means p<0.01 in TTEST. Plants were grown under LD. (TIF) [file pone.0087544.s005.tif]
