# Supplementary material for: The B-Box Family Gene STO (BBX24) in Arabidopsis thaliana Regulates Flowering Time in Different Pathways
Source: PLoS One. 2014 Feb 3;9(2):e87544. doi: 10.1371/journal.pone.0087544 (PMC3911981; doi:10.1371/journal.pone.0087544)
Supplement: Table S1 — Seed list. (PDF) [file pone.0087544.s006.pdf]

Table S1: Seed list

Seeds		
flc-3/FRI/Sf2	from Caroline Dean lab	Nature. 2011 Jul 24;476(7358)
FRI/Sf2	from Caroline Dean lab	Proc Natl Acad Sci U S A. 2007 Feb 27;104
fld-3	from Caroline Dean lab	Plant Physiol. 2003 Jun;132(2):1107-14
sto-1	SALK_067473	
