# Supplementary material for: The B-Box Family Gene STO (BBX24) in Arabidopsis thaliana Regulates Flowering Time in Different Pathways
Source: PLoS One. 2014 Feb 3;9(2):e87544. doi: 10.1371/journal.pone.0087544 (PMC3911981; doi:10.1371/journal.pone.0087544)
Supplement: Table S2 — Primer list. (PDF) [file pone.0087544.s007.pdf]

Table S2: Primer list

**qPCR Primer**	**5'-3'**
UBQ10 :	Ubq s5`-GTACTTTGGCGGATTACAACATC-3`
	Ubq a5`-GAATACCTCCTTGTCCTGGATCT-3`
ACT2	Act s 5`-GGTAACATTGTGCTCAGTGGTGG-3`
	Act a 5`-AACGACCTTAATCTTCATGCTGC-3`
STO	STO F：AATCCACAAGCCAGCAGCAA
	STO R：CCGAAGAATCCCATGTCTGAAAA
STH	STH F：ATCCTCTTAAAATCTCATAAAGCCCTTC
	STH R：TCAGCCTTTTGTTTCTTTCCCTTGT
FLC	sense FLC F：AGCCAAGAAGACCGAACTCA
	sense FLC R：TTTGTCCAGCAGGTGACATC
FLD	FLD F:CGAGCGAACTGGTCGCAAGC
	FLD R:TGCTTCAGCGGCAACGGTCC
FRI	FRI F:TAACGGCGTTGTCCTCGCCG
	FRI R:ACGGTTGGCTCACAGTGACGG
CO	CO F:GAGAAATCGAAGCCCGAGGAGCA
	CO R:TCAGAATGAAGGAACAATCCCATA
FCA	FCA F:GCAAAGAGACGGCAATGGCAGC
	FCA R:GCCATGTCCCTTGACTCGCCA
FT	FT F:ACCTCAGGAACTTCTATACTTTGG
	FT R:TACTATAGGCATCATCACCGTTCG
SOC1	GTGATCTCCACTCAACAAAAA
	CAACAAGAGAGAAGCAGCTTTA
CCA1	CCA1 F:TCCAGATAAGAAGTCACGCTCA
	CCA1 R:TCTAGCGCTT GACCCATAGC
AP1	AP1 F: GCAAGCAATGAGCCCTAAAGAG
	AP1 R: AGTGCGGATGTGCTTAAGAGC
