# Supplementary material for: The B-Box Family Gene STO (BBX24) in Arabidopsis thaliana Regulates Flowering Time in Different Pathways
Source: PLoS One. 2014 Feb 3;9(2):e87544. doi: 10.1371/journal.pone.0087544 (PMC3911981; doi:10.1371/journal.pone.0087544)
Supplement: Table S3 — Rosette leaf number at flowering time for the indicated genotypes. (PDF) [file pone.0087544.s008.pdf]

Table S3: Rosette leaf number at flowering time for the indicated genotypes

Plants were grown under LD (16 h light/8 h dark) and SD (8 h light/16 h dark) conditions. *n*=plant number. (**: TTEST P<0.01)

Line	Rosette leaf number	SD	Range	*n*
Col LD	11.9	±1.1	10-14	28
sto-1 LD	12.8	±1.1	11-14	20
STO-OE LD	7.6 **	±1.0	6-9	28
Col SD	40.5	±2.4	36-43	14
sto-1 SD	50.8 **	±3.1	44-55	20
STO-OE SD	29.0**	±2.8	26-31	22
flc-3/FRI LD	12.5	±1.0	10-14	13
STO-OE x flc-3/FRI-4 LD	7.9 **	±1.0	6-9	10
STO-OE x flc-3/FRI-15 LD	9.5 **	±1.3	8-11	13
FRI SD	84.5	±2.6	81-90	12
flc-3/FRI SD	53.2 **	±1.5	51-56	12
STO-OE x flc-3/FRI-4 SD	43.7 **	±7.8	28-54	9
STO-OE x flc-3/FRI-15 SD	40.7 **	±5.0	31-49	11
STO-OE x FLC/FRI-11 SD	83.8	±2.0	81-87	10
